# Does the creation of healthy cities promote municipal solid waste management? Empirical research in 284 cities in China

**DOI:** 10.3389/fpubh.2022.1030283

**Published:** 2022-10-31

**Authors:** Qingshan Ma, Yutong Zhang, Amoah Samual, Feng Hu, Mohcine Touns

**Affiliations:** ^1^School of Economics, Xiamen University, Xiamen, China; ^2^School of Economics, Jilin University, Changchun, China; ^3^Institute of Digital Economy and Green Development, Chifeng University, Chifeng, China

**Keywords:** Healthy China, Healthy Cities pilot program, municipal solid waste management, domestic pollution, DID

## Abstract

In the context of the COVID-19 pandemic, the creation of healthy cities has become an important measure to deal with global public diseases and public health emergencies, and has had a profound impact on the management of municipal solid waste (MSW). This study exploits the Healthy Cities pilot (HCP) program established in 2016 as a natural experiment, and evaluates its impact on MSW management using the difference-in-difference (DID) method. The estimates show that the collection amount and harmless treatment capacity of MSW were increased by 15.66 and 10.75%, respectively, after the cities were established as pilot healthy cities. However, the harmless treatment rate was decreased by 3.544. This conclusion remains valid in a series of robustness tests, including parallel trend test, placebo test, propensity score matching (PSM)-DID, eliminating the interference of other policies, and eliminating the non-randomness of the policy. Mechanism analysis shows that the HCP program increased the collection amount and harmless treatment capacity of MSW by increasing the expenditure on MSW treatment. However, after a city was established as a pilot healthy city, the unsustainable high expenditure of local government on municipal sanitation led to the decrease in the harmless treatment rate of MSW. Moreover, heterogeneity analysis shows that the HCP program had a stronger impact on MSW management in cities with higher administrative levels, more obvious location advantages, and a larger size. Therefore, it is advisable to use the creation of healthy cities as an important tool to gradually improve MSW management, so as to realize the coordinated development of city construction and human health.

## Introduction

The continued spread of COVID-19 not only poses a huge challenge to global public health security (1–3), but also has a great negative impact on the global economic and social development (4–6). Although the pandemic has been effectively controlled through the implementation of strong containment measures, such as lockdowns (7–9), the shortcomings of public health still remain to be addressed. According to data from the Seventh National Population Census of China, the urban population accounts for 63.89% of its total population. The rapid growth of urbanization has promoted China's economic development, but the resulting “urban disease” has seriously threatened people's life and health (10). According to relevant data from the National Bureau of Statistics of China and the Statistical Yearbook of Urban and Rural Construction, in 2021, the discharge of municipal solid waste (MSW) in China rise rapidly, with the amount of bulky waste reaching 10.8 million tons and that of kitchen waste reaching 255.7 million tons. The decay of MSW breeds bacteria and emits foul odors, which seriously threatens public health (11). The improvement of MSW management is of great significance to improving the city's grade and the living environment of people (12–15).

To solve the increasingly serious problem of domestic pollution, the Chinese government proposed the “Healthy China” strategy, trying to build a sound public health system. The Healthy Cities pilot (HCP) program covering 38 pilot cities^1^ has become an important tool for the implementation of the Healthy China 2030 plan (16, 17). HCP program are all over the east, middle and west of China, which also shows that the establishment of healthy city pilot projects is random to some extent. The pilot cities integrated the concept of health into the whole process of city planning, construction, and management. The questions to be answered in this study are: Can the HCP program promote MSW management? Is there heterogeneity in its impact on MSW management? What are the mechanisms through which the HCP program affects MSW management?

There are two categories of studies related to our work. The first category focuses on the implementation of healthy cities. The second category focuses on how to improve the public health environment of cities. The first category of studies examine the connotation of a healthy city, its metrics, and its impact. The World Health Organization (WHO) defined it in 1994 as people living healthy lives. The goal of creating a healthy city is to make people happier (18). Metrics for evaluating the healthy city development level is an essential part of creating healthy cities. Proper metrics can not only comprehensively measure the health level of a city, but also represent the_connotation of a healthy city (19). In 1996, the WHO suggested 32 indicators for the evaluation of healthy cities. Based on this indicator, relevant scholars established six first-level indicators and used entropy to measure the development level of healthy cities in China (16). Regarding the impact of healthy cities, Harpham et al. (20) used the interview method to evaluate the impact of the HCP program in developing countries, and discovered that these programs were not well-implemented in developing countries as compared with the WHO vision. Yue et al. (21) selected 15 healthy cities using stratified and systematic sampling, and found that the HCP program significantly improved the urban environment based on a DID model. The Healthy Cities initiative promotes the improvement of public services through incentive mechanisms (22) and strengthens inter-sectoral cooperation through competition mechanisms (23). Moon et al. (24) evaluated the policy effects of the implementation of healthy cities in South Korea using a capacity mapping tool, and found that healthy cities played a positive role in the healthy development. In addition, a range of studies have shown the impact of a healthy city environment on improving individual mental health and elderly health (25, 26).

The second category of studies focuses on how to improve the public health environment of cities. Implementing pilot policies is main means of addressing pollution problems in China. First, many studies have focused pilot policies on environmental pollution, and almost all of them reached positive conclusions, that is, the implementation of pilot policies has significantly improved the urban environment. And most of them focus on the control and treatment of industrial pollution sources. For example, Huo et al. (27) and Qiu et al. (28) found that low-carbon city pilot program has a positive impact on the improvement of environmental pollution. Christensen et al. (29) and Song et al. (30) examined the impact of smart city pilot policies on the urban environmental pollution, and found that the creation of smart cities has a positive impact on the healthy and sustainable development of cities. Cao et al. (31) also paid attention to the impact of the implementation of e-commerce demonstration cities on urban environmental pollution, and found that the development of e-commerce is conducive to promoting green transformation. Li et al. (32) studied the impact of carbon emission trading pilot on urban green development, and found that carbon emission trading pilot significantly promoted urban green and healthy development. However, there are relatively few studies that have evaluated healthy city pilot implementation. Second, some studies focus on the control and treatment of domestic pollution sources. Deng et al. (33) examined the impact of “beautiful villages” and “waste sorting” pilot programs in the Yellow River Delta using structural equation modeling, and found that they reduced rural MSW pollution, leading to a reduction of organic matter by 12.1% and nitrogen pollution by 79.8%, thereby comprehensively controlling the spread of domestic pollution sources. Guo et al. (34) overviewed the management of immobilized waste in China from 2004 to 2019, pointed out that changing the industrial structure will change the treatment of immobilized waste, and found that the reduction of solid waste, that of energy consumption, and that of carbon emissions go hand in hand.

To sum up, the existing literature provides many useful leads for examining the impact of the HCP program on MSW management. However, there are still some deficiencies. First, the existing literature on healthy cities (16, 34) mostly uses theoretical research methods and focuses on the connotation and measurement of healthy cities. And no consensus has been reached. Second, previous studies that examined the impact of the creation of healthy cities use either the interview method or the stratified and systematic sampling method (21), and do not use empirical methods. Moreover, most of these studies focus on the impact on the overall ecological environment of the city, and do not consider the impact of the HCP program on MSW management. This provides the motivation for this study to evaluate the Healthy Cities policy using the DID approach, which is widely used in policy evaluation (35, 36). Lastly, most of the literature on the improvement of living environment in cities focuses on the impact of other pilot policies on industrial pollution sources (27, 30–32). However, little effort is made to examine the impact of pilot policies on domestic pollution sources, and even less attention has been paid to the impact of the HCP program on MSW management. Therefore, on the one hand, the policy effects of the HCP program needs to be further evaluated by scientific methods, e.g., DID. On the other hand, further efforts are needed to understand how to control domestic pollution and improve MSW management.

This study provides the following marginal contributions. First, in a theoretical sense, previous studies have paid sufficient attention to the effects of different types of environmental regulations, and the Chinese government has often used pilot policies as a policy vehicle for environmental regulations to study their effects on solving environmental pollution problems, but with mixed environmental praise and criticism. In this paper, we use DID model to assess the impact of HCP program on MSW, which enriches and extends the literature related to the study of policy effects of environmental regulation theory. Second, in terms of research perspective, previous studies have paid sufficient attention to the management of industrial pollution sources (30, 37, 38), whereas little literature directly addresses the management of domestic pollution. Moreover, previous studies have adequately evaluated other pilot policies in cities (27–29, 39), but little attention has been paid to the impact of the creation of healthy cities. This study expands the literature by quantitatively evaluating the impact of the creation of healthy cities on domestic pollution control. Third, in terms of research methods, this study identifies the relationship between healthy cities and MSW management based on the policy shock of the creation of healthy cities using the DID model, which is widely used in policy evaluation (35, 36). This method reduces the error caused by the measurement of Healthy Cities indicators (16, 21). Moreover, a range of robustness tests are performed to ensure valid results. Fourth, to clarify the mechanisms by which the HCP program affects MSW management, this study empirically investigates the “short-term increase in waste management inputs” and “unsustainable long-term high expenditure on municipal sanitation” after the establishment of the pilot healthy cities. This effort provides insights for a better understanding of the operation of healthy cities (16, 40). Fifth, on the impact on public health, this paper provides a realistic basis for further leveraging the HCP program to improve the capacity of MSW management, and then improve the overall public health of the city. For a long time, the implementation of healthy cities has received mixed reviews (24). The findings of this study show that it promoted the collection and harmless treatment of MSW in the short term. However, the unsustainable high expenditure on municipal sanitation in the long run has led to a decline in the harmless treatment rate of MSW. Therefore, future efforts should be made to leverage healthy cities to achieve sustainable and effective MSW management.

## HCP program and research hypothesis

### Introduction to HCP program

A healthy city is a new model of urban development that focuses on human health in all aspects from urban planning, construction to management, intending to ensure healthy living and work for the general public, and organically combining healthy people, a healthy environment and a healthy society. The selection criteria of healthy cities include “healthy environment,” “healthy society,” “healthy services,” “healthy people,” and “healthy culture” 5 primary indicators, 20 secondary indicators, and 42 tertiary indicators. The specific content of each indicator is shown in Supplementary Table 1. In 2016, the National Health Commission of China issued the “Notice on the Pilot Work of Healthy Cities” to launch the pilot creation work of “healthy cities” and continuously improve the policy system by creating a working pattern of healthy city construction with the leadership of the party committee, government leadership, multi-departmental cooperation, support of professional institutions and participation of the whole society. Moreover, the Chinese government has proposed the “6+X”^2^ construction model to encourage each local area to explore some construction models suitable for local conditions. In addition, the Chinese government is also actively guiding the integration of the HCP program with other related efforts, such as integrating the construction of HCP program with maternal and child health promotion and cancer prevention and treatment initiatives.

**Figure 1:**
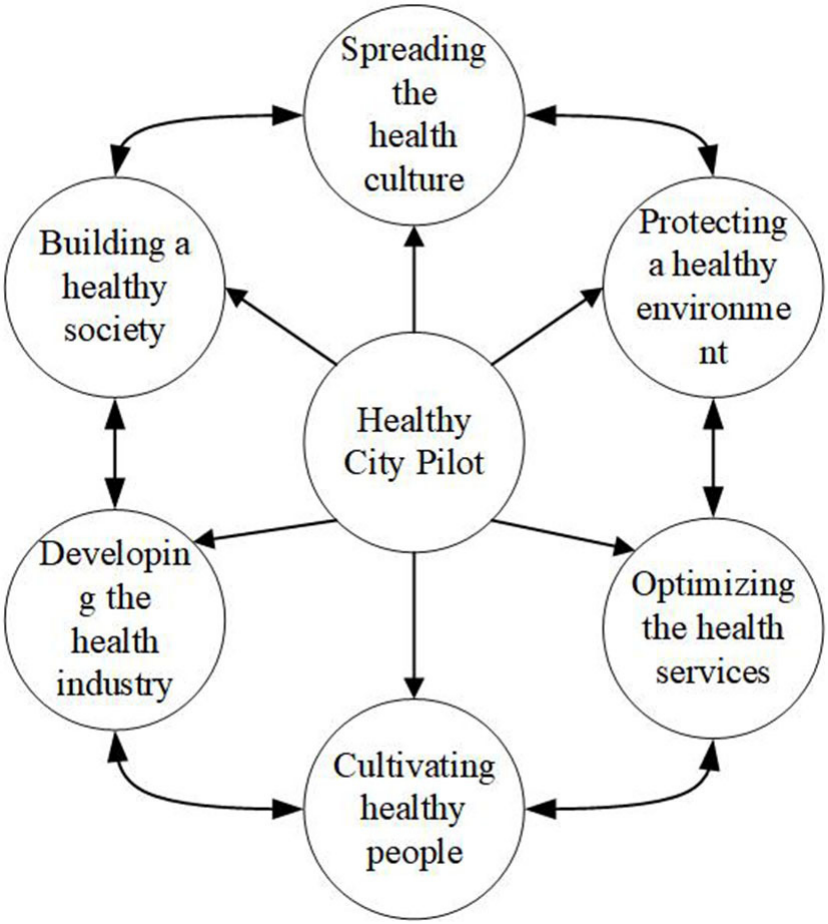
Operation mechanism of a healthy city.

Pilot areas have formed a large number of experiences that can be replicated after a period of construction. For example, by promoting the combination of medicine and physical exercise, organizing health lectures, and promoting MSW management, the average life expectancy in the Xicheng District of Beijing reached 84.26 years in 2018, and health literacy increased from 33.4% in 2015 to 36.6% in 2018. Hangzhou has established six special teams for building a healthy society, spreading the health culture, protecting a healthy environment, optimizing health services, cultivating healthy people, and developing the health industry, as shown in Figure 1.

### Research hypothesis

The HCP program construction will have an impact on MSW management. First of all, the National Healthy City Evaluation Index System (2018 Edition) takes the harmless domestic waste treatment rate as an important element of the HCP program evaluation index system, which has a certain “Target Leading Effect” and “Binding Effect” on the HCP program. The construction of HCP program will focus on a series of construction activities around the harmless removal and treatment of domestic waste, which in turn will affect MSW management. Secondly, the HCP program is led by the party committee, government-led, and multi-departmental cooperation. As an important symbol of healthy city development, local governments often tend to invest money and technology in the construction of healthy cities in order to ensure the smooth implementation of healthy city work. As an important part of HCP program, MSW management will be influenced by the strong drive of local governments. Finally, under China's political system, the central government directly decides the promotion of local government officials, and the HCP program is implemented by the central government, which has the attribute of “strong leadership,” and local governments are often motivated to build HCP program under the drive of the central government. Therefore, as an important policy of the Healthy China 2030 plan, the HCP program has had an impact on MSW management. The HCP program affects MSW management through the following mechanisms.

The HCP program promotes the increase in the amount of MSW collected by increasing related inputs. Guo et al. (34) believed that the treatment of solid non-waste materials and MSW is impossible without capital investment. The HCP program increases the capital and labor input of local governments in waste collection and transportation (41). For example, as an attempt at fine management and operations in municipal sanitation, the method of road cleaning has been changed from sweeping with a broom to vacuum sweeping in the pilot areas. The pilot healthy cities also carried out the “toilet revolution” and the 100-day campaign for environmental sanitation improvement by increasing the purchase of sanitation facilities. According to relevant data of Ma'anshan Civilization Network (mas.wenming.cn), Ma'anshan, as a pilot healthy city, has increased the inputs in waste collection and transportation, so that nearly 280,000 tons of waste are collected and transported annually, and all waste is transported on the day it is produced. The HCP program also encourages the public to participate in it. In addition to advocating for adopting a healthy lifestyle, saving resources, and protecting the environment, it also encourages the public to actively participate in waste collection, resulting in a significant increase in the amount of waste collected. In addition, the HCP program has also improved the capacity for harmless waste treatment by building more waste treatment plants. Also in Ma'anshan, 31 MSW transfer stations and 1 MSW incineration power plant were built, which greatly improved the capacity for harmless waste treatment. Hence, the following hypothesis is proposed:

**H1:** The HCP program improves MSW management by increasing the inputs.

**Figure 2:**
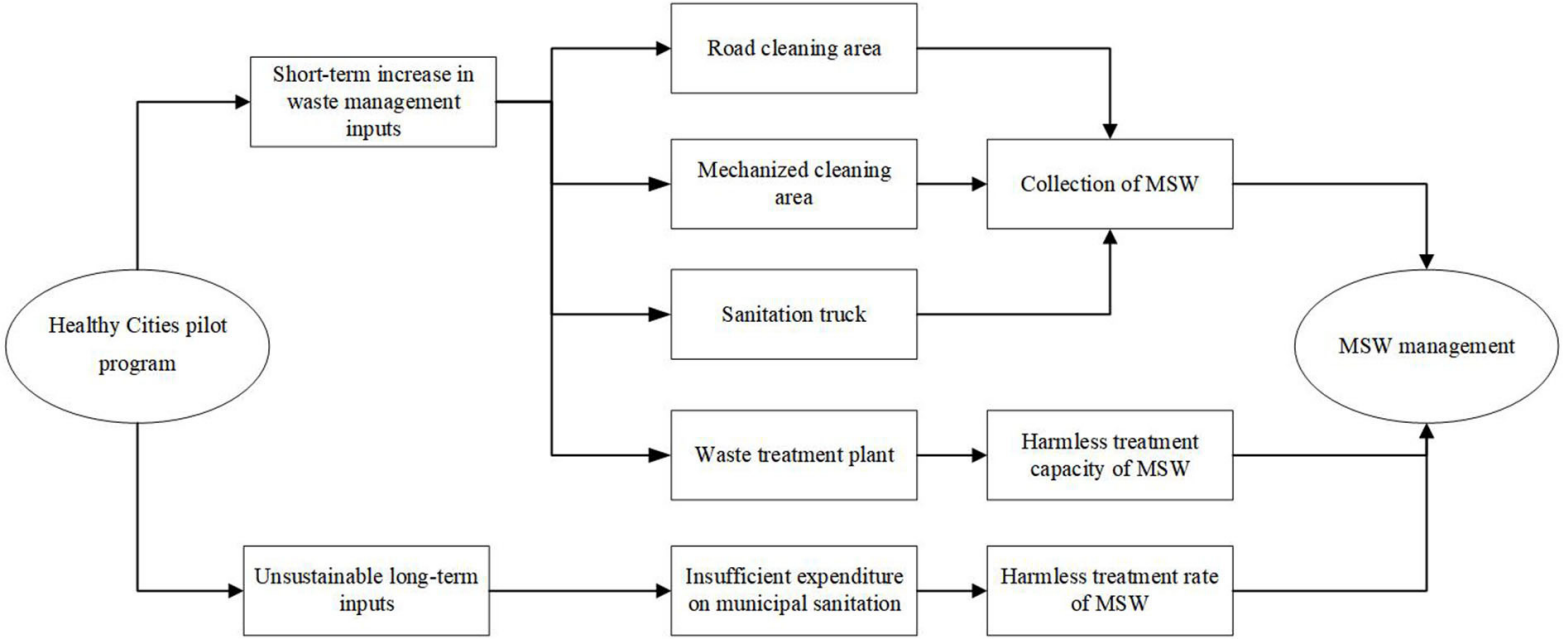
Mechanism structure.

The high expenditure on municipal sanitation may be unsustainable after a city is established as a pilot healthy city, which will lead to a decrease in the harmless treatment rate of MSW. As a typical civic calling card, Healthy Cities are affected by population aging and urban shrinkage in the process of operation and implementation. As a result, local governments will reduce expenditures on public programs, such as the Healthy Cities Initiative (41), which in turn makes the high expenditure on municipal sanitation established in the initial stage of the pilot program unsustainable in the later stage. The overlap between the HCP program and other similar pilot programs may also lead to financial distress for local governments. For example, the demonstration zone for comprehensive prevention and treatment of chronic diseases launched in China in 2010 coincides with the HCP program in terms of living environment improvement (6). In addition, some alienation behaviors may interfere with the proper operation of the pilot program. For example, small restaurants never provide paper napkins to consumers to build a healthy environment. Accordingly, the mechanical structure of this paper is shown in Figure 2, and the following hypothesis is proposed:

**H2:** During the implementation of the HCP program, the unsustainable high expenditure on municipal sanitation may compromise MSW management.

## Methodology and data

### Methodology

We evaluate the impact of healthy city implementation on MSW management using the DID model. According to Duflo (36), the following DID model is constructed:


(1) $$\begin{array}{l} Yit=\beta 0+\beta 1Hea\text{-}City_{it}+\gamma Control_{it}+\mu i+\nu t+\varepsilon it \end{array}$$

where Y_it_ is the dependent variable which represents the amount of MSW collected (Rem-vol), the harmless treatment capacity of MSW (Tre-cap), and the harmless treatment rate (Tre-rate) of the city *i* in year *t*. Hea-City_it_ is the core independent variable which is 1 for the year when a city was selected as a pilot healthy city and the subsequent years, and 0 for the other years. Its coefficient β1 reflects the impact of healthy city pilot implementation on municipal sanitation improvement. *X* is a set of control variables, including financial pressure (fin-pressure), industrial structure (ind-stru), population density (pop-density), level of opening to the outside world (opening), science expenditure (sci-expe), financial development level (fin-deve), and economic development level (lnpergdp). μ*i* is a city fixed effect that controls for the characteristics of a city that do not change over time. ν*t* is a year fixed effect that controls for the macro policy shocks faced by cities. ε*it* is a random disturbance term. This model effectively controls for the characteristic differences between the experimental and control groups and the time variation trend.

### Data and variables

The dependent variable is MSW management (Y_it_), which is measured by the amount of MSW collected (Rem-vol), the harmless treatment capacity of MSW (Tre-cap), and the harmless treatment rate (Tre-rate). The amount of MSW collected is the amount of MSW collected and transported to MSW treatment plants (in 10,000 tons). It is expressed in logarithms in the empirical analysis. The main methods of harmless treatment of MSW are sanitary landfill, high-temperature composting, and incineration by using advanced technology to minimize the environmental harm of MSW. The harmless treatment capacity of MSW refers to the amount of MSW that can be harmlessly treated by waste treatment plants per day (in ton/day). It is expressed in logarithms in the empirical analysis. The harmless treatment rate of MSW is the ratio of the amount of MSW treated harmlessly to the amount generated. However, the amount of MSW generated is often replaced by the amount of MSW collected due to data unavailability. The data are from the *China Urban and Rural Construction Statistical Yearbook*.

The core independent variable is the pilot healthy cities (Hea-City_it_). The pilot healthy cities are identified according to the list of the first batch of 38 pilot healthy cities approved by the National Health Commission of China (www.nhc.gov.cn) in 2016.

To address the problem of omitted variables, other factors that affect municipal sanitation are also controlled for. The pressure of financial revenue and expenditure directly determines the capacity of domestic waste cleaning and transportation; Opening to the outside world can enable us to learn from the world's advanced experience in treating domestic waste; The important role of technological progress has been confirmed by relevant literature (42). The level of scientific expenditure will affect the mechanized disposal rate of domestic waste; The higher the level of financial development, the more special funds available for domestic waste treatment. The level of economic development is also an important factor affecting the treatment of MSW. In addition, the greater the proportion of the secondary industry and the population density, the more domestic waste emissions. Therefore, we selected the following control variables. The control variables include: financial pressure (fin-pressure), measured by (financial expenditure—financial revenue)/financial revenue; industrial structure (ind-stru), measured by the ratio of the added value of the secondary industry to GDP; population density (pop-density), measured by total population/area; level of opening to the outside world (opening), measured by foreign direct investment (FDI)/GDP; science expenditure (sci-expe), measured by total science expenditure/GDP; financial development level (fin-deve), measured by (year-end deposit balance + year-end loan balance)/GDP; and economic development level (lnpergdp), measured by per capita GDP in logarithm.The data are from the China City Statistical Yearbooks. The descriptive statistics for each variable are shown in Table 1.

**Table 1:** Descriptive statistics.

**Variables**	**Observations**	**Mean**	**Std. dev**.	**Min**	**Max**
Rem-vol	4,504	3.3598	0.9292	−0.6931	6.9189
Tre-cap	3,978	6.8115	0.9841	0.6931	10.5978
Tre-rate	4,024	93.1573	15.0232	0	100.0001
pop-density	4,544	428.1758	332.134	−19.27	2,661.54
ind-stru	4,544	47.0361	11.2365	9	90.97
opening	4,496	27.7937	40.2937	−169.9393	1,215.717
sci-expe	4,544	22.1676	25.0536	0.0003	630.9981
fin-pressure	4,544	1.9133	2.4962	−0.3512	96.8512
fin-deve	4,544	22700	12,000	0.0819	213,000
lnpergdp	4,544	1.1676	0.8376	−1.4084	3.9747
Rsa	4,504	6.8538	1.0529	3.2581	11.6217
San-veh	4,498	5.1133	1.2688	1.0986	9.5032
Msr	4,221	0.4655	0.2609	0	1
San-per	4,535	8.8161	0.7856	−0.844	11.683
Ghtp	4,008	1.986	2.4594	0	43
Inpl	1,732	1.2881	1.3872	0	12
Sltp	3,647	1.3861	1.4333	0	21
Hea-exp	3,918	8.6797	1.7375	1.9459	14.0109

## Empirical results

### Baseline regression results

We first investigate the impact of the HCP program on MSW management. The regression results of model (1) are shown in Table 2. No control variables are included in columns (1), (3), and (5). Control variables that affect MSW management are included in columns (2), (4), and (6). The region fixed effect (city) and the time fixed effect (year) are controlled for in columns (1)–(6). The regression results in columns (2), (4), and (6) are analyzed as an example. The HCP program significantly improved the amount of MSW collected (by 15.66%) and the harmless treatment capacity of MSW (by 10.75%), but reduced the harmless treatment rate of MSW (by 3.544). The possible reason for this is as mentioned above; that is, the HCP program may increase the collection amount and harmless treatment capacity of MSW by increasing the waste management inputs of local governments; and the decrease in the harmless treatment rate of MSW may be due to insufficient expenditure on municipal sanitation.

**Table 2:** Baseline regression.

	**(1)**	**(2)**	**(3)**	**(4)**	**(5)**	**(6)**
	**Rem-vol**	**Rem-vol**	**Tre-cap**	**Tre-cap**	**Tre-rate**	**Tre-rate**
Hea-City	0.1426^***^	0.1566^***^	0.5938^***^	0.1075^**^	−4.0653^***^	−3.5446^**^
	(0.0536)	(0.0438)	(0.0462)	(0.0503)	(1.4072)	(1.3712)
pop-density		0.0004^**^		0.0002		−0.0134^***^
		(0.0002)		(0.0002)		(0.0045)
ind-stru		−0.0032		−0.0149^***^		0.0747
		(0.0023)		(0.0019)		(0.1023)
opening		−0.0008^*^		−0.0007		0.0314^***^
		(0.0005)		(0.0006)		(0.0097)
sci-expe		0.0019^***^		0.0012^*^		−0.0405^**^
		(0.0007)		(0.0007)		(0.0191)
fin-pressure		−0.0037		0.0048		0.7898^**^
		(0.0078)		(0.0042)		(0.3126)
fin-deve		−0.0000		0.0000		0.0000
		(0.0000)		(0.0000)		(0.0000)
lnpergdp		0.3791^***^		0.4868^***^		−3.3601
		(0.0774)		(0.0327)		(2.9934)
Constant	3.2429^***^	3.2097^***^	6.7847^***^	6.7359^***^	77.8446^***^	78.2210^***^
	(0.0268)	(0.1253)	(0.0021)	(0.1505)	(1.5351)	(5.0841)
Observations	4,504	4,456	3,978	3,933	4,024	3,979
Adjusted R^2^	0.2362	0.2873	0.0500	0.3897	0.2396	0.2547

Numbers in brackets are robust standard errors clustered at the city level.

***, **, and * indicate p < 0.01, p < 0.05, and p < 0.1, respectively.

Controlled for year fixed effects and city fixed effects.

### Parallel trend test

A crucial assumption for the validity of the DID is the parallel trend assumption. Moreover, considering that the effect of the creation of pilot healthy cities on MSW management may be delayed and dynamic, we test for parallel trends and dynamic effects using the event study methodology based on the baseline regression model (35). The econometric model is shown in Equation (2), where k represents the k*th* year of healthy city pilot implementation. The maximum value of k is 4, and the minimum value is −11 during the sample period of this study (2005–2020). For presentation purposes, relative times < −5 are merged into −5. Moreover, it is often necessary to define a base period to avoid multicollinearity when using the event study methodology to test for parallel trends and dynamic effects. The year before healthy city pilot implementation (−1) is selected as the base period. Then, the values of k are −5, −4, ... −2, 0, ...4. The coefficient ofβ*k* represents the difference in MSW management between the experimental and control groups in the k*th* year of healthy city pilot implementation compared with the year before the implementation.


(2) $$\begin{array}{l} Yit=\beta 0+\beta k\overset{k=\text{4}}{\underset{k=\text{-5}}{\sum }}Hea\text{-}City_{it}^{k}+\gamma Control_{it}+\mu i\\ \;+\nu t+\varepsilon it \end{array}$$

**Figure 3:**
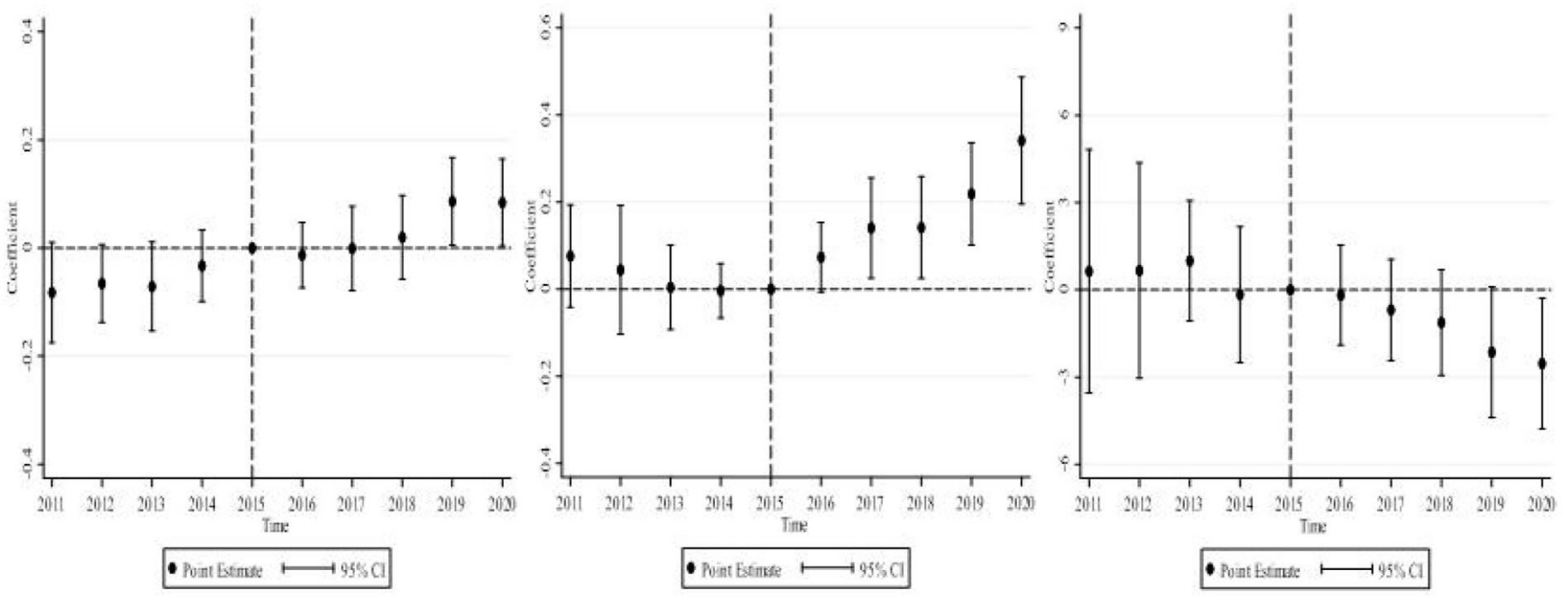
Parallel trends and dynamic effects.

The results of the tests are shown in Figure 3. The three graphs from left to right show the results obtained by taking the amount of MSW collected, the harmless treatment capacity of MSW, and the harmless treatment rate of MSW as dependent variables, respectively. All the results show that there were no significant differences in MSW management between the experimental and control groups before the healthy city pilot implementation. It indicates that our DID satisfies the parallel trend assumption. Estimates of dynamic effects show that MSW management was not immediately improved at the beginning of healthy city implementation. Therefore, the HCP policy had a delayed effect. As the policy continued to be implemented, its effect on increasing the collection amount and harmless treatment capacity of MSW gradually emerged, but it reduced the harmless treatment rate of MSW. It is foreseeable that with the further implementation of the policy, it will have a more pronounced effect on MSW management.

### Robustness tests

#### Placebo test

**Figure 4:**
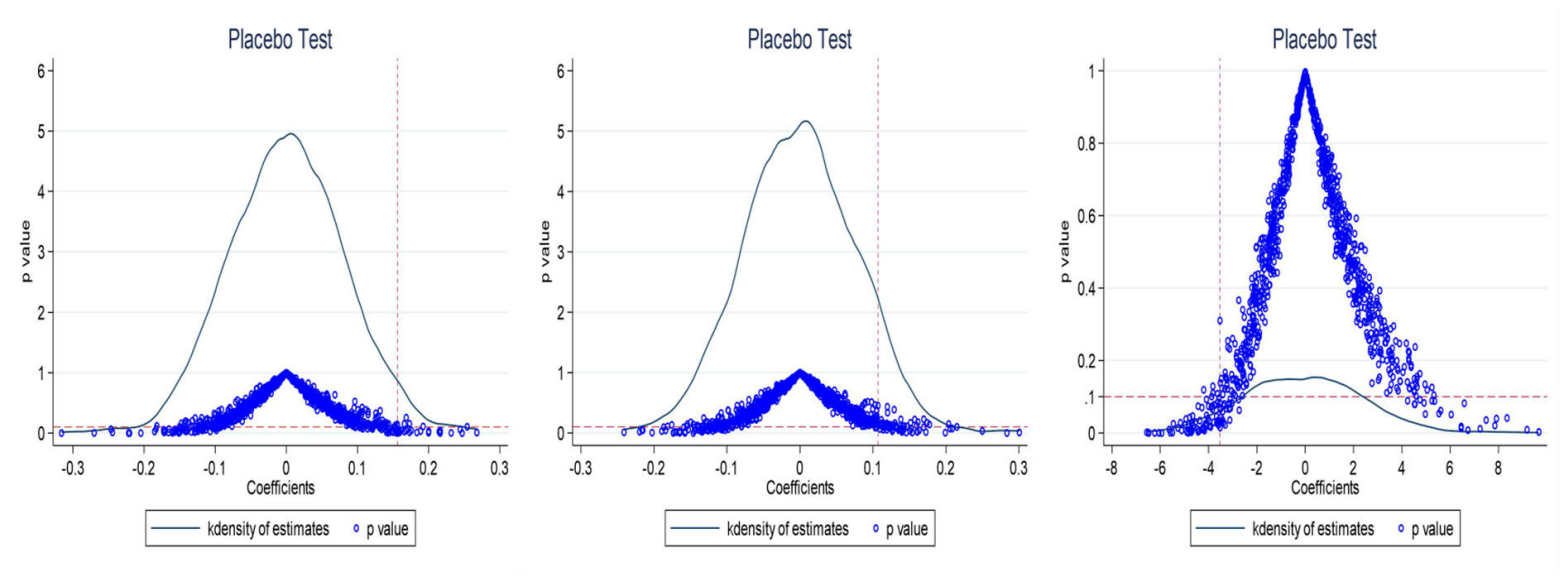
Placebo test.

Another concern with using the DID model to assess the effect of healthy city implementation on MSW management is that the conclusions obtained may be a random phenomenon. To exclude the influence of non-observed factors, this paper uses the method of Li et al. (43) for reference to carry out the placebo test. Specifically, we randomly select some cities as experimental groups using Stata, and then randomly assign a time to each experimental group selected as the policy start time. Finally, a “fictitious” policy variable is introduced as an independent variable for regression with the dependent variable. This process is repeated 1,000 times. The coefficients from 1,000 regressions and the corresponding *p*-values are plotted in the same graph, and compared with the coefficients obtained from the baseline regression. The three graphs in Figure 4 show the results of 1,000 regressions obtained by taking the amount of MSW collected, the harmless treatment capacity of MSW, and the harmless treatment rate of MSW as dependent variables, respectively. The coefficients of 1,000 regressions with a dummy policy variable are concentrated around 0, and the *p*-values for most of the regressions are > 0.1, indicating that the regression results are not significant. This further demonstrates that HCP program implementation significantly increased the collection amount and harmless treatment capacity of MSW, but reduced the harmless treatment rate of MSW. The above conclusions are robust.

#### Propensity score matching DID

Although the DID model can scientifically identify the average treatment effect of the pilot policy, it may lead to a significant selection effect considering that the establishment of pilot healthy cities may not be completely random. Therefore, PSM-DID is used for robustness testing according to Heckman et al. (44). Generally speaking, PSM is only used for cross-sectional data, while the DID is used for panel data. We use two methods for matching. The first method treats the sample data as cross-sectional data for mixed matching. The second method is period-by-period matching according to Bockerman and Ilmakunnas (45).

**Figure 5:**
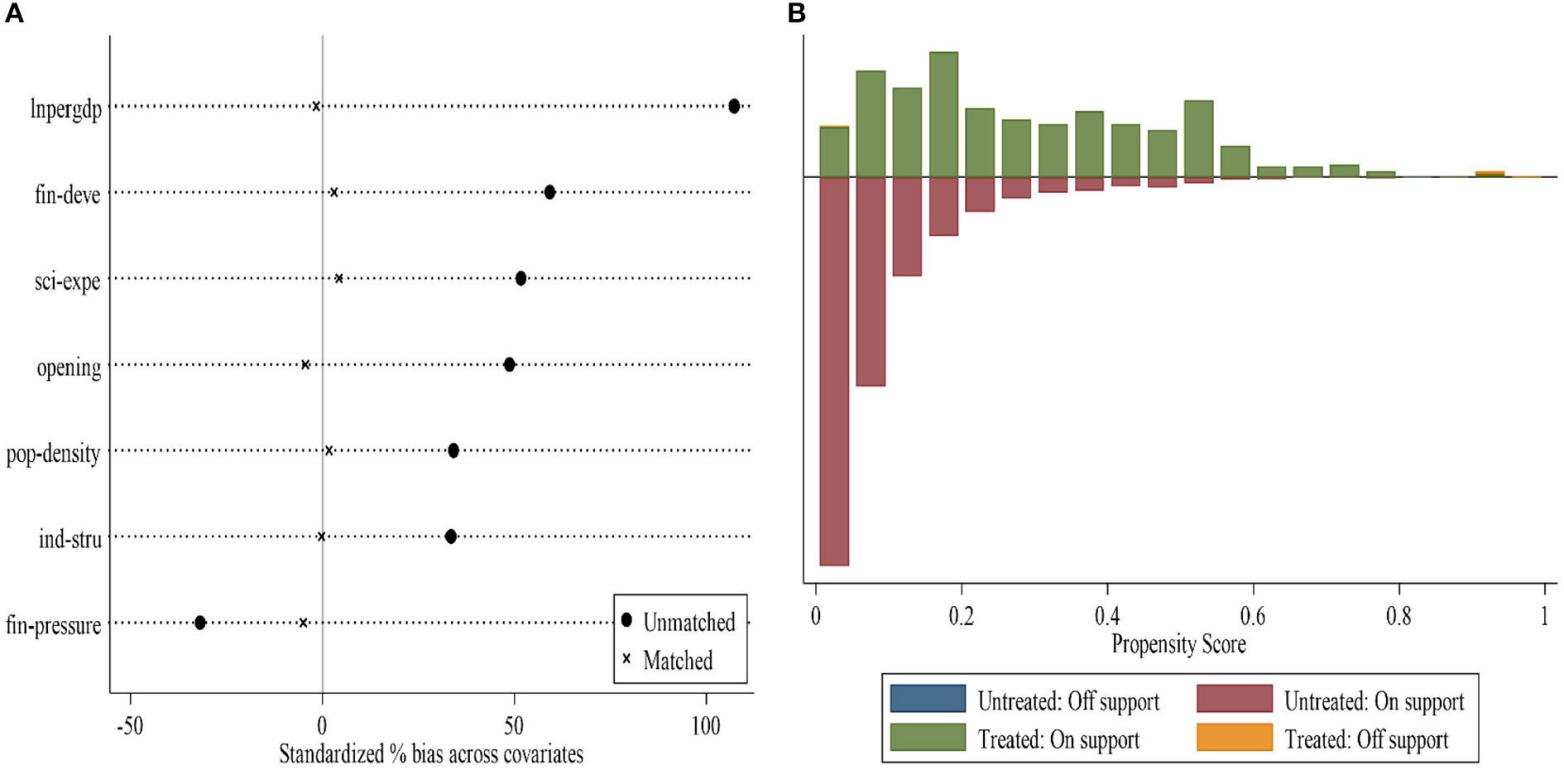
**(A,B)** Balance test.

**Figure 6:**
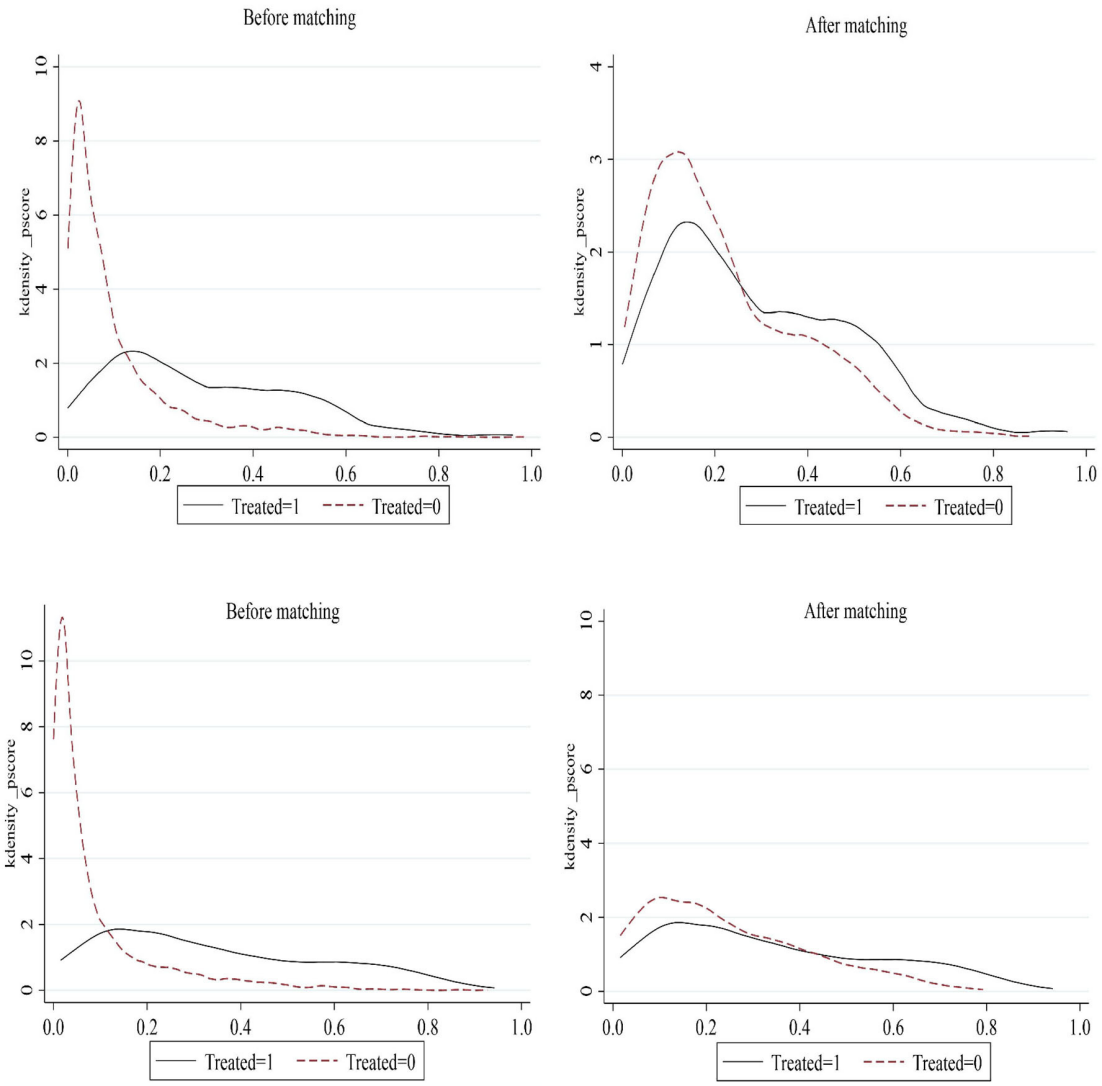
Matching test.

The steps are as follows: First, financial pressure, industrial structure, population density, level of opening to the outside world, science expenditure, financial development level, and economic development level are used as matching variables. Second, two new data sets are obtained using the two matching methods, for both of which nearest neighbor matching is used. Next, the balance of the two data sets is tested and the data distribution before and after matching is compared. The results of the balance test are shown in Figure 5. It can be seen that the standardized deviations of most variables significantly decrease after matching, and most of the observations are within the common value range, indicating that it is reasonable to use the PSM-DID method. As shown in Figure 6, we found that the average fitting water of the experimental group and the control group was better after the two methods were matched. Therefore, the PSM method adopted in this paper is more effective. Finally, the DID is used to perform the same two-way fixed effects estimation as for the baseline regression on each of the two data sets. The regression results of PSM-DID are presented in Table 3. Columns (1)–(3) present the regression results obtained using cross-sectional data matching. Columns (4)–(6) present regression results obtained using period-by-period matching. It can be seen that the healthy city pilot implementation significantly increased the collection amount and harmless treatment capacity of MSW, but reduced the harmless treatment rate of MSW.

**Table 3:** PSM-DID regression results.

	**(1)**	**(2)**	**(3)**	**(4)**	**(5)**	**(6)**
	**Rem-vol**	**Rem-vol**	**Tre-cap**	**Tre-cap**	**Tre-rate**	**Tre-rate**
Hea-City	0.1479^***^	0.0999^*^	−3.5130^**^	0.0792^*^	0.0974^*^	−2.4088^**^
	(0.0430)	(0.0514)	(1.3757)	(0.0448)	(0.0569)	(1.2267)
Control variable	Yes	Yes	Yes	Yes	Yes	Yes
Constant	3.2771^***^	6.6665^***^	78.4071^***^	3.4477^***^	6.6603^***^	82.1402^***^
	(0.1263)	(0.1596)	(5.2696)	(0.1385)	(0.1954)	(3.8590)
Observations	4,447	3,925	3,972	3,023	2,715	2,743
Adjusted R^2^	0.2952	0.3935	0.2548	0.4294	0.4059	0.2338

Numbers in brackets are robust standard errors clustered at the city level.

***, **, and * indicate p < 0.01, p < 0.05, and p < 0.1, respectively.

Controlled for year fixed effects and city fixed effects.

#### Eliminating interference of other policies

Although the HCP program significantly affects domestic pollution, it is undeniable that there are other pilot policies in the same period that may also affect MSW management. For example, the pilot programs of civilized city and low-carbon city (28) have an effect on the conclusions of this study. We further control for the dummy variables of whether the city was established as a pilot civilized city and a pilot low-carbon city in the relevant year based on the baseline regression. The estimates are shown in Table 4. The robustness of the results estimated in this paper after excluding the relevant policy disturbances.

**Table 4:** Eliminating interference of other policies.

	**(1)**	**(2)**	**(3)**
	**Rem-vol**	**Tre-cap**	**Tre-rate**
Hea-City	0.1274^***^	0.0856^*^	−2.8850^**^
	(0.0435)	(0.0507)	(1.3834)
Low-carbon city	0.0848^***^	0.0813^*^	−1.3843
	(0.0325)	(0.0424)	(1.5220)
Civilized city	0.1500^***^	0.1176^***^	−4.5468^***^
	(0.0329)	(0.0410)	(1.0462)
Control variable	Yes	Yes	Yes
Constant	3.1776^***^	6.7137^***^	78.9160^***^
	(0.1193)	(0.1495)	(4.9338)
Observations	4,456	3,933	3,979
Adjusted R^2^	0.3043	0.3954	0.2626

Numbers in brackets are robust standard errors clustered at the city level.

***, **, and * indicate p < 0.01, p < 0.05, and p < 0.1, respectively.

Controlled for year fixed effects and city fixed effects.

#### Eliminating non-randomness of the policy

The establishment of pilot healthy cities may be affected by the inherent characteristics of the cities and thus is not completely random. According to Duflo (36), we add the interaction of a confounding variable S_i_ with the year fixed effect ν*t* to the traditional DID model to minimize the non-randomness of the establishment of pilot healthy cities. Specifically, two categories of variables are considered. The first category of variables is natural geographical characteristics (Nature) of the city, such as river density, elevation, and slope. The reason is that natural geographical factors affect vegetation growth, and to a certain extent, the health level of the city (46), which may affect whether the city is established as a pilot healthy city. The second category of variable is social and cultural characteristics (Society) of the city, such as education, which is measured by the number of full-time teachers in regular institutions of higher education, cultural level, which is measured by the number of books in public libraries, and pollution, which is measured by sulfur dioxide emissions. As shown in columns (1)–(6) of Table 5, the HCP program increased the collection amount and harmless treatment capacity of MSW, but reduced the harmless treatment rate of MSW. This proves once again that the above regression results are robust.

**Table 5:** Eliminating non-randomness of the policy.

	**(1)**	**(2)**	**(3)**	**(4)**	**(5)**	**(6)**
	**Rem-vol**	**Rem-vol**	**Tre-cap**	**Tre-cap**	**Tre-rate**	**Tre-rate**
Hea-City	0.1439^***^	0.0717^*^	0.1056^**^	0.1131^*^	−3.6101^***^	−3.0329^**^
	(0.0434)	(0.0419)	(0.0522)	(0.0628)	(1.3820)	(1.3276)
Natural^*^ν*t*	Yes	Yes	Yes	Yes	Yes	Yes
Society^*^ν*t*	No	Yes	No	Yes	No	Yes
Control variable	Yes	Yes	Yes	Yes	Yes	Yes
Constant	4.9558	13.2149	−43.4630^***^	−44.6826^***^	101.9994	−76.8636
	(15.9574)	(16.2245)	(15.9642)	(16.9405)	(534.1019)	(573.1415)
Observations	4,440	3,608	3,925	3,122	3,972	3,162
Adjusted R^2^	0.3160	0.3068	0.4101	0.3580	0.2556	0.2318

Numbers in brackets are robust standard errors clustered at the city level.

***, **, and * indicate p < 0.01, p < 0.05, and p < 0.1, respectively.

pControlled for year fixed effects and city fixed effects.

## Further discussion

### Mechanism analysis

The estimates in this study indicate that the HCP program significantly increased the collection amount and harmless treatment capacity of MSW, but reduced the harmless treatment rate of MSW. Next, we analyze the mechanisms by which healthy city implementation affects MSW management.

#### Mechanism for increasing the amount of MSW collected

As the HCP program continues to be implemented, the amount of MSW collected has increased significantly. The possible reason is that the local government has increased the capital and labor input in MSW collection after the city was established as a pilot healthy city. Accordingly, we take the road cleaning area (Rsa), mechanized cleaning rate (Msr), and number of municipal sanitation trucks (San-veh) as the capital input of the local government in MSW collection, and the number of municipal sanitation workers (San-per) as the labor input. As shown by the regression results in Table 6, the HCP program significantly increased the local government's labor and capital input in MSW collection. Specifically, the healthy city pilot implementation increased the road cleaning area by 24.24%, the mechanized cleaning rate by 7.79%, the number of sanitation trucks by 29.39%, and the number of municipal sanitation workers by 19.92%.

**Table 6:** Mechanism for healthy city implementation to increase the amount of MSW collected.

	**(1)**	**(2)**	**(3)**	**(4)**
	**Rsa**	**Msr**	**San-veh**	**San-per**
Hea-City	0.2424^***^	0.0779^***^	0.2939^***^	0.1992^***^
	(0.0375)	(0.0179)	(0.0561)	(0.0269)
Control variable	Yes	Yes	Yes	Yes
Constant	6.3737^***^	0.7080^***^	4.9090^***^	8.5246^***^
	(0.0817)	(0.0414)	(0.1224)	(0.0587)
Observations	4,456	4,184	4,450	4,487
Adjusted R^2^	0.3448	0.3953	0.3394	0.2835

Numbers in brackets are robust standard errors clustered at the city level.

*** Indicate p < 0.01, p < 0.05, and p < 0.1, respectively. Controlled for year fixed effects and city fixed effects.

#### Mechanism for increasing the harmless treatment capacity of MSW

The most direct mechanism for healthy city implementation to increase the harmless treatment capacity of MSW is the construction of more waste treatment plants. The *China Urban and Rural Construction Statistical Yearbook* includes the number of MSW harmless treatment plants (Ghtp), that of landfill plants (Sltp), and that of incineration plants (Inpl). They are used as dependent variables for regression, respectively. As shown by the regression results in Table 7, the HCP program significantly increased the number of MSW harmless treatment plants and incineration plants. However, it did not increase the number of landfill plants. The possible reason is that landfill requires a lot of land resources, but cities in China have suffered from land shortage as a result of increasing urbanization.

**Table 7:** Mechanism for healthy city implementation to increase the harmless treatment capacity of MSW.

	**(1)**	**(2)**	**(3)**
	**Ghtp**	**Inpl**	**Sltp**
Hea-City	1.4858^***^	1.2781^***^	0.1419
	(0.4972)	(0.4688)	(0.2627)
Control variable	Yes	Yes	Yes
Constant	0.7953	0.9829	0.9171^***^
	(0.6699)	(0.5997)	(0.3216)
Observations	3,963	1,723	3,602
Adjusted R^2^	0.2561	0.2901	0.0271

Numbers in brackets are robust standard errors clustered at the city level.

*** Indicate p < 0.01, p < 0.05, and p < 0.1, respectively. Controlled for year fixed effects and city fixed effects.

#### Mechanism for decreasing the harmless treatment rate of MSW

**Figure 7:**
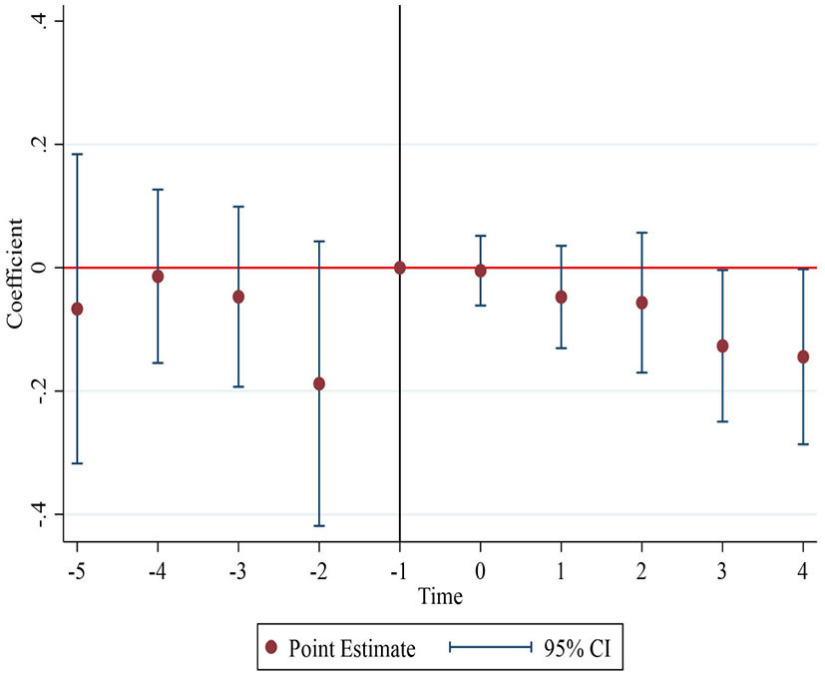
Changes in expenditure on municipal sanitation.

As the HCP program continues to be implemented, the harmless treatment rate of MSW has decreased significantly. According to the definition, the harmless treatment rate of MSW is the ratio of the amount of MSW treated harmlessly to the amount collected. The above conclusions of this study indicate that the HCP program increased the amount of MSW collected. Then the decrease in the harmless treatment rate of MSW is due to the fact that the increase in the amount of MSW treated harmlessly (numerator) is smaller than the increase in the amount of MSW collected (denominator). However, as indicated above, the HCP program significantly increased the harmless treatment capacity of MSW. Therefore, the smaller increase in the amount of MSW treated harmlessly is unlikely to be due to poor equipment for MSW harmless treatment, but probably due to the fact that the local government reduced the expenditure on MSW harmless treatment, leading to increased equipment idleness of MSW treatment plants. To test this conjecture, we examine the impact of the HCP program on local government's expenditure on municipal sanitation (Hea-exp). As shown by the regression results in Figure 7, the expenditure on municipal sanitation decreased sharply after 3 years of the pilot implementation. It shows that the local government's high expenditure on municipal sanitation is unsustainable as the HCP program continues to be implemented.

### Heterogeneity analysis

#### Heterogeneity by city level

The effect of healthy city implementation on MSW management may vary with the administrative level of cities. Accordingly, provincial capitals, municipalities directly under the central government, cities specifically designated in the state plan, and special economic zones are defined as cities with high administrative levels, and other cities are defined as cities with low administrative levels. Cities with high administrative levels are assigned a value of 1, and those with low administrative levels are assigned a value of 0 to generate the Rank variable. By introducing the interaction (Rank^*^Hea-City) between the dummy variable (Rank) for city level and the dummy variable (Hea-City) for the HCP policy into the baseline regression, a two-way fixed effects DID regression is performed to examine the difference in the effect of the HCP program on MSW management due to the different administrative levels of cities. Table 8 reports the regression results, and we find that the impact of MSW management in the HCP is more pronounced in cities with higher administrative levels. The possible reason is that compared with ordinary cities, provincial capitals, municipalities directly under the central government, cities specifically designated in the state plan, and special economic zones are often the core cities in China's regional economic development. They have unique advantages in terms of economic foundation and public health resources, which offer synergy with the HCP program. By contrast, healthy city implementation has a smaller effect on the improvement of MSW management in ordinary cities due to a less developed concept of healthy living and the constraints of GDP growth goals.

**Table 8:** Heterogeneity by city level.

	**(1)**	**(2)**	**(3)**
	**Rem-vol**	**Tre-cap**	**Tre-rate**
Rank^*^Hea-City	0.2218^***^	0.1249^**^	−2.8005^*^
	(0.0513)	(0.0560)	(1.6281)
Control variable	Yes	Yes	Yes
Constant	3.2301^***^	6.7573^***^	77.6996^***^
	(0.1242)	(0.1490)	(3.0597)
Observations	4,456	3,933	3,979
Adjusted R^2^	0.2875	0.3892	0.2534

Numbers in brackets are robust standard errors clustered at the city level.

***, **, and * indicate p < 0.01, p < 0.05, and p < 0.1, respectively.

Controlled for year fixed effects and city fixed effects.

#### Heterogeneity by city location

The effect of healthy city implementation on MSW management may vary with the location of cities. Accordingly, the sample cities are divided into eastern, central, and western cities by geographic location. Western cities are assigned a value of 1, central cities are assigned a value of 2, and eastern cities are assigned a value of 3 to generate the Location variable. By introducing the interaction (Location^*^Hea-City) between Location and Hea-City into the baseline regression, a two-way fixed effects DID regression is performed to examine the difference in the effect of the HCP program on MSW management due to the different locations of cities. As shown by the regression results in Table 9, the effect of Location^*^Hea-City on MSW collection is significantly positive at the 5% level, indicating that healthy city implementation has a more pronounced effect on increasing MSW collection in eastern cities with obvious location advantages. Likewise, the effect of Location^*^Hea-City on the harmless treatment capacity of MSW is significantly positive at the 1% level, indicating that healthy city implementation has a more pronounced effect on increasing the harmless treatment capacity of MSW in eastern cities with obvious location advantages. However, the effect of Location^*^Hea-City on the harmless treatment rate of MSW is negative at the 5% level, indicating that healthy city implementation has a more pronounced negative effect on the harmless treatment rate of MSW in eastern cities with obvious location advantages. The possible reason is that compared with the central and western cities with fewer location advantages, the eastern cities have higher technological levels, better economic foundation, and a more mature concept of healthy living, which provide a good foundation for healthy city pilot implementation, thus allowing a greater improvement in MSW management.

**Table 9:** Heterogeneity by city location.

	**(1)**	**(2)**	**(3)**
	**Rem-vol**	**Tre-cap**	**Tre-rate**
Location^*^Hea-City	0.0585^**^	0.0439^*^	−1.4248^**^
	(0.0226)	(0.0254)	(0.5908)
Control variable	Yes	Yes	Yes
Constant	3.2205^***^	6.7465^***^	77.9672^***^
	(0.1254)	(0.1501)	(3.0610)
Observations	4,456	3,933	3,979
Adjusted R^2^	0.2848	0.3892	0.2540

Numbers in brackets are robust standard errors clustered at the city level.

***, **, and * indicate p < 0.01, p < 0.05, and p < 0.1, respectively.

Controlled for year fixed effects and city fixed effects.

#### Heterogeneity by city size

The effect of healthy city implementation on MSW management may vary with the size of cities. Accordingly, the sample cities are divided into large and small cities according to the total population. Specifically, cities with a greater than average population are assigned a value of 1, and those with a smaller than average population are assigned a value of 0 to generate the Scale variable. By introducing the interaction (Scale^*^Hea-City) between Scale and Hea-City into the baseline regression, a two-way fixed effects DID regression is performed to examine the difference in the effect of the HCP program on MSW management due to the different sizes of cities. As shown by the regression results in Table 10, the effect of Scale^*^Hea-City on MSW collection is significantly positive at the 1% level, indicating that healthy city implementation has a more pronounced effect on increasing MSW collection in larger cities. Likewise, the effect of Scale^*^Hea-City on the harmless treatment capacity of MSW is significantly positive at the 1% level, indicating that healthy city implementation has a more pronounced effect on increasing the harmless treatment capacity of MSW in larger cities. However, the effect of Scale^*^Hea-City on the harmless treatment rate of MSW is negative at the 1% level, indicating that healthy city implementation has a more pronounced negative effect on the harmless treatment rate of MSW in larger cities. The possible reason is that compared with small cities, large cities have better conditions for MSW treatment, thus allowing a greater improvement in MSW management.

**Table 10:** Heterogeneity by city size.

	**(1)**	**(2)**	**(3)**
	**Rem-vol**	**Tre-cap**	**Tre-rate**
Scale^*^Hea-City	0.1871^***^	0.1435^***^	−3.9736^***^
	(0.0449)	(0.0493)	(1.3414)
Control variable	Yes	Yes	Yes
Constant	3.2159^***^	6.7385^***^	78.0544^***^
	(0.1259)	(0.1501)	(5.0757)
Observations	4,456	3,933	3,979
Adjusted R^2^	0.2873	0.3901	0.2545

Numbers in brackets are robust standard errors clustered at the city level.

*** Indicate p < 0.01, p < 0.05, and p < 0.1, respectively.

Controlled for year fixed effects and city fixed effects.

## Conclusions and public health implications

This study regards the HCP program implemented as a policy shock of the creation of healthy cities, and evaluates the effect of the HCP program on MSW management using. The results showed that the HCP program promoted a 15.66% increase in the amount of MSW collected, and a 10.75% increase in the harmless treatment capacity of MSW, but resulted in a reduction of 3.544 in the harmless treatment rate. Mechanism analysis shows that the HCP program promoted the collection and harmless treatment capacity of MSW by increasing the short-term inputs in MSW treatment. However, insufficient expenditure on municipal sanitation was the main reason for the decrease in the harmless treatment rate of MSW after a city was established as a pilot healthy city. Moreover, heterogeneity analysis shows that the HCP program had a stronger impact on MSW management in cities with higher administrative levels, more obvious location advantages, and a larger size.

The implications of this paper for public health research are as follows. First, the continued spread of COVID-19 poses a huge challenge to global public health security and economic and social development, and China's public health and medical security system is in urgent need of upgrading. In this paper, we found that HCP program has a strong positive impact on MSW management and has positive implications for the construction of the public health system. Therefore, we can use the HCP program as an important “grasp” for public health improvement, summarize the successful experience of healthy city construction, and promote it in China and around the world. Secondly, this paper finds that although the HCP program can promote MSW management through high investment in the short term, it still faces the dilemma of declining support funds in the long term. Therefore, the construction of a public health system requires a long-term process, and attention should be paid to the sustainability and long-term nature of the public health policy represented by HCP program. Finally, the heterogeneity study found that there are some differences in the effect of the HCP program, so the construction of the public health system should be mainly different.

## Implications, limitations, and directions for further research

Some policy implications can be drawn from this study. First, the scope of the HCP program should be further expanded. The government should summarize the experience of pilot cities in promoting healthy living, waste management, and environmental pollution control in a timely manner, and expand the scope of the HCP program, thereby providing lessons for the nationwide implementation of Healthy Cities. Second, the policy effects of the HCP program should be promoted from multiple perspectives. It is found that as the HCP program continues to be implemented, the unsustainable expenditure of local governments on municipal sanitation has led to a decline in the harmless treatment rate of MSW. Therefore, it is necessary to coordinate the HCP program and other pilot programs (i.e., low-carbon cities, civilized cities, and innovative cities) to ensure the sustainability of local financial support for public health improvement. Moreover, efforts should be made to maximize the coordination and linkage of the HCP program with other public health policies. Finally, the HCP program should be implemented according to local conditions. In ordinary cities, cities with fewer location advantages, and small cities, efforts should be made to create an institutional environment that is compatible with the operation of Healthy Cities to promote its effective operation. Cities with high administrative levels, obvious location advantages, and a large size should gain the first-mover advantage and maintain the sustainability of municipal sanitation improvement as pilot healthy cities.

This study has two limitations. First, medical waste is an important part of MSW. The collection and harmless treatment capacity of the medical waste directly affect the efficiency of MSW management, especially in the context of the global spread of COVID-19. However, due to the unavailability of medical waste data, this study cannot evaluate the impact of the HCP program on the collection and harmless treatment of medical waste. Second, this study finds that the HCP program has a significant positive impact on MSW management in the short term, but does not assess its impact on people's health—both physical and mental.

Based on the existing limitations, future research can consider the following: First, field surveys can be conducted to collect data on the production, collection, and harmless treatment capacity of medical waste in each city. The impact of the creation of healthy cities on the collection and harmless treatment capacity of medical waste can then be evaluated using the DID method based on the survey data. Second, the impact of the HCP program on the healthy growth of adolescents, people's mental health, and the health of the elderly can be evaluated using micro-survey databases to fully understand the direct impact on people.

## Data availability statement

The original contributions presented in the study are included in the article/Supplementary material, further inquiries can be directed to the corresponding author.

## Author contributions

All authors were involved in the research, writing, proofreading of this paper, and confirmed the final version.

## Conflict of interest

The authors declare that the research was conducted in the absence of any commercial or financial relationships that could be construed as a potential conflict of interest.

## Publisher's note

All claims expressed in this article are solely those of the authors and do not necessarily represent those of their affiliated organizations, or those of the publisher, the editors and the reviewers. Any product that may be evaluated in this article, or claim that may be made by its manufacturer, is not guaranteed or endorsed by the publisher.

## Footnotes

1 The 38 healthy city pilots specifically include: Xicheng District of Beijing, Heping District of Tianjin, Qian'an, Houma, Baotou, Dalian, Changchun, Daqing, Jiading District of Shanghai, Suzhou, Wuxi, Zhenjiang, Hangzhou, Ningbo, Tongxiang, Ma'anshan, Xiamen, Yichun, Jinan, Weihai, Yantai, Zhengzhou, Yichang, Zixing, Zhuhai, Nanning, Qionghai, Hechuan District of Chongqing, Chengdu, Luzhou, Guiyang, Yuxi, Lhasa, Baoji, Jinchang, Golmud, Yinchuan and Karamay.

2 The “6” is to establish the leadership mechanism of the party committee and government, develop a healthy city development plan, carry out “health cell” construction, promote a number of key construction projects, establish a national health management system, and carry out the evaluation of the construction effect. “X” means to promote the construction of characteristics.
