# Supplementary material for: Does the creation of healthy cities promote municipal solid waste management? Empirical research in 284 cities in China
Source: Front Public Health. 2022 Oct 31;10:1030283. doi: 10.3389/fpubh.2022.1030283 (PMC9659738; doi:10.3389/fpubh.2022.1030283)
Supplement: Supplementary file 1 [file Table_1.DOCX]

**Additional Table 1 |** Healthy city evaluation index system

| **Tier 1 Indicators** | **Secondary indicators** | **Tertiary indicators** |
| --- | --- | --- |
| Healthy Environment | 1.Air Quality | （1）Percentage of days with good ambient air quality |
|  |  | （2）Number of heavily polluted days and above |
|  | 2.Water Quality | （3）Living drinking water quality standard rate |
|  |  | （4）Centralized drinking water source safety and security compliance rate |
|  | 3.Garbage waste disposal | （5）Harmless disposal rate of domestic waste |
|  | 4.Other related environments | （6）Density of public restroom settings |
|  |  | （7）The prevalence of environmentally sound sanitary toilets (rural) |
|  |  | （8）Green space per capita |
|  |  | （9）Vector biological density control level |
|  |  | （10）National sanitary counties (townships) as a percentage |
| Healthy Society | 5.Social Security | （11）Actual reimbursement ratio of basic medical insurance inpatient expenses |
|  | 6.Fitness Activities | （12）Urban sports field area per capita |
|  |  | （13）The ratio of the number of social sports instructors per 1,000 people |
|  | 7.Occupational Safety | （14）Occupational health inspection coverage |
|  | 8.Food Safety | （15）Food Sampling and Inspection |
|  | 9.Cultural Education | （16）Excellent rate of student physical fitness monitoring |
|  | 10.Retirement | （17）Number of elderly beds per 1,000 elderly population |
|  | 11.Health Cell Engineering | （18）Healthy Community Coverage |
|  |  | （19）Healthy school coverage |
|  |  | （20）Healthy Business Coverage |
| Healthy Services | 12.Mental Health Management | （21）Standardized management rate of patients with severe mental disorders |
|  | 13.Maternal and Child Health Services | （22）Child Health Management Rate |
|  |  | （23）Maternal system management rate |
|  | 14.Health Resources | （24）Number of general practitioners per 10,000 population |
|  |  | （25）Number of public health personnel per 10,000 population |
|  |  | （26）Number of beds in medical and health institutions per 1,000 population |
|  |  | （27）Percentage of primary health care institutions providing TCM services |
|  |  | （28）Share of health spending in fiscal spending |
| Healthy People | 15.Health Level | （29）Life expectancy per capita |
|  |  | （30）Infant mortality |
|  |  | （31）Under-five mortality rate |
|  |  | （32）Maternal mortality rate |
|  |  | （33）The proportion of urban and rural residents who passed the National Physical Fitness Standards |
|  | 16.Infectious Diseases | （34）Incidence of Class A and B infectious diseases |
|  | 17.Chronic diseases | （35）Premature mortality from major chronic diseases |
|  |  | （36）Prevalence of hypertension among 18-50 year olds |
|  |  | （37）Magnitude of change in age standardized incidence of tumors |


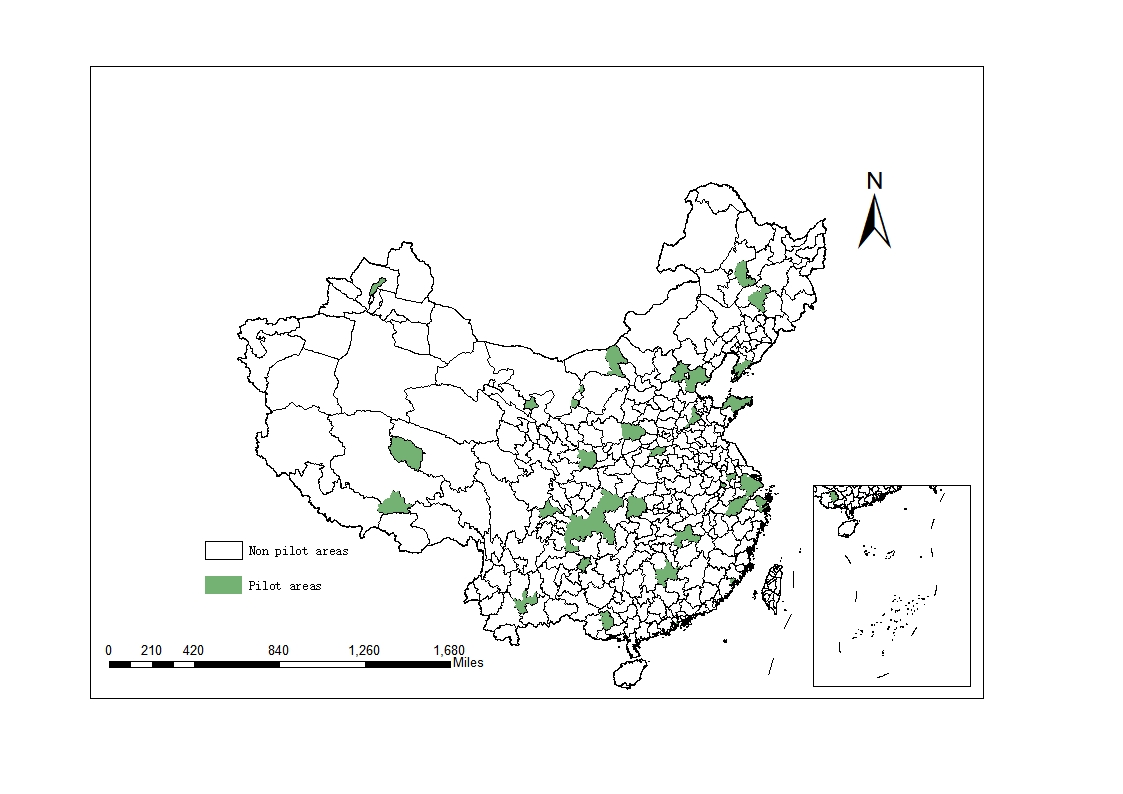


**Additional Figure 1** | Schematic diagram of pilot area
